# 

**Published:** 1887-02-19

**Authors:** 


The Hospital.

An Institution, Family, and Parochial Journal of Hospitals, Asylums, and all Agencies for the Care of the Sick, Criticism and News.

SATURDAY, FEBRUARY 19TH, 1887.

### The Literary Prize.

A PRIZE of **Two Guineas** will be given every month for the best story or incident based upon hospital, or asylum, or student life, or any incident connected with the professions of medicine or nursing.

### The Quilter Prizes.

### HOSPITAL HOUSEKEEPING AND MANAGEMENT.

We offer two prizes of Ten Guineas and Five Guineas respectively for the two best reports on the Expenditure of any single hospital, such reports to give all the details concerning the cost of food and stimulants, brought out in detail in the Table on page ii of the Special Supplement we published on February 5th, together with a critical examination of the various recommendations contained in the Report of the Special Committee of the Newcastle Infirmary, and the addition of the figures to the following heads of expenditure. Hospital secretaries may procure a printed form of return, containing all the particulars to be filled up by competitors, on application to the Editor.

1. *Domestic Expenses.*—Soap and Soda; Laundry, Repairs, and Washing; Water Rate; Coals, Coke, and Wood; Gas and Oil; Bedding and Linen; Furniture, Earthenware, and Hardware; Uniform and Livery, for (*a*) Porters, (*b*) Sisters and Nurses.

2. *Surgery and Dispensary.*—Drugs; Surgical Dressings and Bandages; Instruments, Artificial Limbs, and Trusses; Mineral Waters; Dispensary Sundries.

3. *Salaries and Wages.*—(*a*) Management, including Secretary, Superintendent, Clerks, and Collectors (including commission); (*b*) Nursing Department, Matron or Lady Superintendent, Superintendents of Nursing, Sisters, Nurses, Extra Nurses, Ward Maids and Scourers; (*c*) Maintenance of Porters and Servants (not included in *a* and *b*).

4. *Management Expenses.*—Printing and Stationery; Advertisements; Postage; Special Appeal for Funds; Annual Court and Festival Dinner; Auditor's Fee; Banker's Interest.

5. *Rates and Insurance* (exclusive of Water and Gas rates).

6. *Pensions*, showing to which Department they belong.

7. *Repairs and alterations of Structur*.—(*a*) General, including flooring and painting; (*b*) Extraordinary, including building.

8. *Miscellaneous Expenses* (*i.e.*, all other items to be given in detail).

All the figures are to be those for the year 1886, and the totals must agree with the figures published in the annual report, and copy of which must be sent in with the manuscript. Competitors must enclose their name and address, must write on one side of the paper only, and give the name of the hospital they have selected. No competition should occupy more space than that represented by three pages of printed matter in The Hospital.

All competitions must be sent in not later than the 31st of March next, addressed to the Editor, The Hospital, Norfolk House, Norfolk Street, London, W.C., and it is hoped that particulars may in this way be supplied from every British Hospital.

### THE CHILDREN'S CORNER.

### OUR PASTIMES.

### Children's Quarterly Prize Competition.

Began 1st Jan., 1887: ends 26th Mar., 1887.

PRIZES.

FOR MOST CORRECT ANSWERS.

| | | | |
|---|---|---|---|
| 1st . .. | Thirty Shillings | 3rd .. .. | Ten Shillings |
| 2nd .. .. | Twenty ,, | 4th .. .. | Five ,, |

FOR NEATEST ANSWERS.
Five Shillings.

FOR BEST LITTLE LETTERS.
Ten Shillings.

### Eighth Competition.

No. 43. CONUNDRUM.

White as chalk,
Light as down,
Soft as silk,
Moist as froth,
What is it?

No. 44. GEOGRAPHICAL ENIGMA.

In barter, not in sell,
In metal, not in bell,
In accept, not in refuse,
In zouave, not in muse,
In oak, not in ash,
In linen, not in crash,
My *whole* is a river in America.

. . * . .
. * .
*
. * .
. . * . .

No 45. HOUR-GLASS PUZZLE. Copper coins; a word meaning owing the beginning of every *race;* a word meaning to request; a number of ships in company.

No. 46. BURIED TREES. Clara shall see them; My boa keeps me warm; Ethel makes baskets; See, how well I mend nets; Find my pin, Ella; All arches are not beautiful. Find me, if you please, six trees.

### Results of Sixth Competition.

No. 33. CONUNDRUM. Why does Mr. Irving's company at the Lyceum resemble Messrs. Maskelyne and Cooke's entertainment? Because, of course, there is a mystery (Miss Terry) associated with both!

No. 34. DECAPITATION This has puzzled some of my little friends. One tells me that he searched a natural history through, and "beheaded all the birds," but could not find one to meet the conditions. Behead "Plover," and we get Lover, the author of *Handy Andy*.

No. 35. DIAMOND PUZZLE. Everyone right here.

P
r A m
t a S t e
P A S T I M E
s h I n e
A M y
E

No. 36. Everyone also has discovered "Guy's."

*All right*—Skye, A. C. K., Tim; *Three right*—Alsie, Prospect, Mikado, Peeler, Paul Methven; *Two right*—Florrie, P. S., Ellie, Isobel, Scrooge, Dot, Joe, Little Mary, Bismarck, Ariadne, Inez.

### Letters from Little Folk.

Though many of my young friends have returned to school, there is no diminution either in the interest or in the quantity of their correspondence. I think, perhaps, it will be better if, in future, I set the subject of the letters each week, so that they may all be upon the same. Next week I would like a letter about "Books." Tell me whether you like them, the kind you read, and so on. Scrooge writes me about her school:—

DEAR MR. EDITOR,—I will put off writing about the concert I said I would, as you wish me to tell you about my studies. I like going to school very much. Of all my studies I like Geography and Algebra the best, and History the least. I have commenced to learn Latin and French, but I do not like them as much as I thought I should.

Yours truly, SCROOGE.

7, Charles Street, Pendleton, Manchester.

Here is a letter from Skye about the weather:—

DEAR MR. EDITOR,—I can only write a short letter about the weather this time. It is so changeable here in Kent. One day very warm, and the next day biting off our skin with cold. Half of next day is rain, and the other half fog. We have been in the Western Islands and the Orkney Islands in the far North of Scotland. It never was so cold there as here in the South of England, and not at all changeable. We gathered full-blown primroses in February in the Isle of Skye. We are expecting more snow and sledging immediately.—I remain, yours faithfully, SKYE.

4, Milton Terrace, New Road, Chatham.

Peeler sends me the following interesting letter about the Orphanage of Mercy at Kilburn. Charity can take no worthier form than by helping poor children, the innocent victims of so much of this world's suffering:—

DEAR MR. EDITOR,—Perhaps it would interest you to hear what we have been doing with ourselves. Well, a few days ago the magazine from the Orphanage of Mercy arrived here, containing a notice to this effect:—"A Bazaar will be held on the 10th, 11th, and 12th of May, in Willis's Rooms. The receipts of the Bazaar will go towards building our new Orphanage, to be called, in commemoration of the Jubilee, the Queen Victoria Orphanage." Perhaps you will ask, why build a new orphanage? They have got one; what more do they want? Dear Mr. Editor, the reason why is, 1st, that the present Orphanage is as full as it can be; 2nd, that many poor little ones are refused admittance, because there is "no room"; 3rd, what better remembrance of the Jubilee could you have than a home for poor and destitute children? I am afraid this letter is longer than I thought it would be; but I must ask one more question. Will not somebody who has lots of leisure try and see how many articles he can make before the 10th of May? All communications may be sent to Miss Thomas, 27, Kilburn Park Road, N.W. And now, dear Mr. Editor, I really must put an end to this.—Believe me yours truly, PEELER.

Eaton, Chester.

### Letter Prize.

One mark each: H., Tim, Peeler, Florrie, Scrooge, Mikado, Skye, P. S., Ellie, Inez, and Ariadne.

### Notices to Correspondents.

H.—How nice those tricycle rides will be with Alsie. Be sure you let me have an account of the first ride.

MULEY.—Many thanks for contribution. Please send me your full name next time you are writing.

MIKADO.—What an addition to the family you will have, to be sure! Thank you, I shall be glad of any further contributions.

FLORRIE. That was a shocking accident to the little girl, but I am sure everything possible will be done for her at the London Hospital.

---

Answers must be addressed to the "PUZZLE EDITOR," THE HOSPITAL, Norfolk House, Norfolk Street, Strand, W.C., not later than Thursday next. The "Children's Corner" Coupon (see advertisement pages) must be enclosed.

The address and full Christian name and surname, and the age of the competitor, must be stated in all replies. A *nom de plume* may be added for the purposes of publication.

### AMUSEMENTS WITH PROFIT.

### PRIZE COMPETITIONS.

**Quarterly Prize Competition.**

Began 22nd January; Ends 16th April.

**A Prize of Three Guineas**

will be awarded to the Competitor who shall have sent in the largest number of correct or best answers. No one will be permitted to win the Three Guinea Prize twice in any one year, but we shall award annually an additional

**Prize of Five Guineas**

to the competitor who shall have sent in the largest number of correct or best answers during the twelve months.

---

### No. 9. Double Acrostic.

My *primals* the poor in their sickness need;
For my *finals*, my *primals* earnestly plead.
* * * * * *
A *slayer of children*, hated, despised;
A Portuguese *port* for its wine much prized;
A truly wondrous *orb*, thou art;
A fam'd *canal* 'twill knit two oceans great;
A mass of silver in an *unwrought state*,
Points where all *trains* arrive, depart;
In Corse, the town of a *great soldier's* birth;
The *king of beasts* acknowledged o'er the earth;
Found in the *wheels* of ev'ry cart.

### No. 10. Arithmetical Question.

A countrywoman, carrying eggs to a garrison, where she had to pass three guards, sold at the first guard half the number she had and half an egg more; at the second, half of what remained, and half an egg more, and at the third, half of what remained, and half an egg more. When she arrived at the market-place she had still three dozen eggs to sell. How was it possible without breaking any of the eggs, and how many had she originally?

### Answers.

No. 5. DOUBLE ACROSTIC.

S ig H

T rur O

H our S

O utstri P

M ena I

A rara T

S al A

S au L

Correct answers have been received from Patient, Paddy, Ellice, C. le Maitre, Mrs. Bedingfeld, H. B., Ostrich, Condorcet, Frederick C., Gretta, Mr. J. High, Tabs, Mater, Perseverance, A. M. E., H. J. C., K. M.

No. 6. DEFINITION OF "HOSPITAL."

Most of the definitions sent in by our competitors have been in verse. Some of the answers display much epigrammatic power and fertility of thought.

Best answers have been received from Paddy, Ellice, Patient, H. B., Ostrich, Condorcet, Frederick C., Gretta, K. M., Tabs, Mater, Perseverance, A. M. E., H. J. C.

### Notices to Correspondents.

PEELER.—You had two "lights" wrong in No. 5.

A. M. E.—Thanks for your letter. We are pleased you like this feature of THE HOSPITAL.

H. J. C.—Your reply to No. 3 was "he himself with you, etc.," which is not correct. Of course you are not "barred" from future competitions. We do not anticipate that any competitor will solve every question correctly.

Answers must be addressed to THE PRIZE EDITOR, AMUSEMENTS WITH PROFIT, THE HOSPITAL, Norfolk House, Norfolk Street, Strand, W.C., not later than Saturday next. The full name and address must in all cases be given, but a *nom de plume* may be added for publication. Only one side of the paper should be written on. The "Amusements with Profit" Coupon (see advertisement pages) must be enclosed with replies.

---

### BUSINESS COMMUNICATIONS AND ADVERTISEMENTS.

All communications concerning business matters, non-delivery of THE HOSPITAL, etc., should be addressed to the Manager, at the Publishing Offices, 30 and 32, Sardinia Street, Lincoln's Inn Fields, London, W.C.

**Cheap Advertisements** of the "Wanted" class, including those for situations, of Twenty-four Words or under, are charged 1*s*. an insertion, or three insertions for 2*s*. 6*d*., but must be paid for in advance. Each additional line of Eight Words is 4*d*.

**Employers requiring assistants** are charged for one insertion of Twenty-four Words or under, 2*s*. 6*d*., or 6*s*. for three insertions. Each additional line of Eight Words is 1*s*.

**Advertisements** for insertion in the weekly issue of THE HOSPITAL **must be delivered** at the Publishing Offices **not later than 11 a.m. on Wednesdays.**

### The In- and Out-Patients' Hospital Diary.

This Diary is so arranged as to make some portion of it appear every week, and the whole in each monthly part. It has been very difficult to compile, and corrections will at all times be gratefully received. It has been suggested that similar information about the larger Provincial and Scotch Hospitals would be useful to many readers. We should be glad to hear if this is felt to be the case.

### CHILDREN'S DISEASES.—*continued.*

| Hospitals and Terms of Admission. | Physicians, Surgeons, and Medical Officers, with Days and Hours of Attendance. |
|---|---|
| Samaritan Free Hospital for Women and Children, Lower Seymour Street, W.; Branch, 1, Dorset St., W. Sec., Mr. G. Scudamore. In-patients must be between 2 and 12. Out-patients Department (free) is at Edward's Mews, Duke Street, W. | *In-Physician*, Dr. W. H. Day. Daily, 2. *In-Surgeon*, Mr. W. A. Meredith. Daily, 2. *Out-Physicians*, Drs. M. Prickett and Amand Routh, W. S. 1.30. *Out-Surgeons*, Messrs. W. A. Meredith and J. D. Malcolm, M. Th.; A. H. G. Doran and W. S. A. Griffith, Tu. F., 1.30 |
| Victoria Hosp. for Children, Queen's Road, Chelsea, S.W. Sec., Capt. W. C. Blount, R.N. Age of boys, 2 to 12 years; age of girls, 2 to 16. Admission for in-patients by subscriber's letter. Accidents and urgent cases free at all times | *In-Physicians*, Drs. J. Evans, M. Th. 2; Ridge-Jones, Tu. F. 2. *In-Surgeons*, Messrs. Pick, Tu. F. 9.15; Clutton, M. Th. 4.30. *Out-Physicians*, Drs. A. Venn, W. S. 9.30; C. Fox, M. Th. 1 30; F. D. Drewitt, Tu. F. 9; J. Philpot, M. Th. 9. *Out-Surgeons*, Messrs. F. Churchill, Tu. F. 10; W. Pye, M. Th. 9.30 |
| West London Hospital, Hammersmith rd, W. | Daily, 2. *See* General Hospitals |

### CONSUMPTION AND DISEASES OF THE CHEST.

| Hospitals and Terms of Admission. | Physicians, Surgeons, and Medical Officers, with Days and Hours of Attendance. |
|---|---|
| City of London Hospital for Diseases of the Chest, Victoria Park, E. Sec., Mr. T. Storrar Smith. By subscriber's letter available for 6 weeks. | *In-Physicians*, Drs. J. Thorowgood, S.; E. Smith, M.; J. Fothergill, W.; S. West, F.; G. A. Heron, W.; V. D. Harris, Tu. Hour, 2. *Out-Physicians*, Drs. V. D. Harris, Tu.; J. A. Ormerod, Th.; E. C. Beale, Tu. F.; B. Fenwick, W. S.; A. Money, W. S.; L. E. Shaw, M. Th. Hour, 2 |
| Hospital for Consumption, Fulham road, Brompton, S.W. Sec., Mr. H. Dobbin. Admission by letter of recommendation, but, where unobtainable, application may be made to the Secretary at the Hospital. | *In-Physicians*, Drs. E. S. Thompson, M. Th.; C. T. Williams, M. Th.; R. D. Powell, Tu. F.; J. Tatham, W. S.; R. E. Thompson, W. S.; F. T. Roberts, Tu. F. Hours, 2 to 4. *In-Surgeon*, Mr. R. J. Godlee, F. *Out-Physicians*, Drs. T. H. Green, J. M. Bruce, M. Th.; J. K. Fowler, W. S.; P. Kidd and C. Y. Biss, Tu. F.; T. D. Acland, W. S. Hour, 12 |
| North London Hospital for Consumption and Diseases of the Chest, Mount Vernon, Hampstead, N. Sec., Mr. L. F. Hill. Admission by subscriber's letter available for 6 weeks. | *In-Physicians*, Drs. A. Evershed, Tu.; B. O'Connor, F.; D. Pidcock, F. Hour, 10.30. *Out-Physicians*, Drs. B. O'Connor, D. Pidcock, J. E. Squire. Daily, 2 (attend at Out-patients' Branch, 216, Tottenham Court Road, W.) |
| Royal Hospital for Diseases of the Chest, 231, City Rd., E.C. Sec., Mr. John J. Austin. By Governor's letter, available for six weeks | *In-Physicians*, Drs. Hensley, W.; Gilbart-Smith, M.; Finlay, Th. *In-Surgeon*, Mr. A. Pearce-Gould, M. Tu. W. Th. F. 2, S. 9. *Out-Physicians*, Drs. White, Tu. F.; Pringle, M. Th.; O. Browne, W. S.; A. Davies, M. F.; H. Habershon, W. S.; E. Stewart, Tu. Th. Hour, 1; S. 9 |
| Royal National Hosp. for Consumption and Diseases of the Chest, Ventnor. Offices, 34, Craven St., Strand, W.C. Sec., Mr. E. Morgan. Admits only incipient cases by Governor's letter. | *In-and Out-Physicians*, Drs. J. G. Sinclair Coghill, and R. Robertson attend daily (at Ventnor) on out-patients at 11; on in-patients at noon. *In- and Out-Surgeons*, Messrs. Whitehead, Williamson |

### EAR CASES.

| Hospitals and Terms of Admission. | Physicians, Surgeons, and Medical Officers, with Days and Hours of Attendance. |
|---|---|
| Central London Throat and Ear Hosp., Gray's Inn Rd., W.C. Sec., Mr. R. Kershaw. Free, but when able, a small payment is expected | *In- and Out-Surgeons*, Mr. Lennox Browne, M, Th. 2.30; Dr. Orwin, W. S. 2.30; Dr. Grant, Tu. 5, Th. 2.30; Messrs. P. S. Jakins, M. W. 2.30; F. 5; T. Carmalt Jones, M. Th. S. 2.30 |
| Charing Cross Hospital, West Strand, W.C. | *In- and Out-Surgeon*, Mr. Cantlie, F. 9 |
| Great Northern Central Hospital, Caledonian Rd, N. | *In- and Out-Surgeon*, Mr. A. E. Cumberbatch, W. 9 |
| Guy's Hospital, St. Thomas's Street, S.E. | *In- and Out-Surgeon*, Mr. Purves, Tu. F. 12.30 |
| Hospital for Diseases of the Throat, Golden Square, W. Sec., Mr. W. T. Sharp | *Physicians*, Drs. Wolfenden, Tu. F. 2.30; Macdonald, Tu. F. 6.30; Bond, W. S. 2.30. *Surgeon*, Mr. T. M. Hovell, M. Th. 2.30 |

### EAR CASES—*Continued.*

| Hospitals and Terms of Admission. | Physicians, Surgeons, and Medical Officers, with Days and Hours of Attendance. |
|---|---|
| King's College Hospital, Portugal Street, W.C. | *In- and Out- Surgeon*, Mr. Pritchard Th. 2.30 |
| London Hospital, Whitechapel Road, E. | *In- and Out-Surgeons*, Dr. Woakes, Mr. T. M. Hovell, S. 9 |
| Middlesex Hospital, Berners Street, W. Free. | *Out-Surgeon*, Mr. Hensman, Tu. 9 |
| National Hosp. for the Paralysed, etc., Queen Sq., W.C. | *In- and Out-Surgeon*, Mr. A. E. Cumberbatch, W. 2 |
| North West London Hospital, 18, Kentish Town Rd. | *Out-Surgeon*, Mr. Collier, M. 1.30 |
| St. Bartholomew's Hospital, Smithfield, E.C. | *Surgeon*, Mr. Cumberbatch, Tu. F. 2 |
| St. George's Hospital, Hyde Park Corner, S.W. | *In- and Out-Surgeon*, Sir W. B. Dalby, Tu. 1.30 |
| St. Mary's Hospital, Cambridge Place, Paddington, W. | *In- and Out-Surgeon*, Mr. G. P. Field, W. S. 9.30 |
| St. Thomas's Hospital, Westminster Bridge Road, S.E. | *Out-Surgeon*, Mr. Clutton, M. 1.30 |
| University College Hospital, Gower Street, W.C. | *In- and Out-Surgeon*, Mr. Barker, Th. 9 |
| Westminster Hospital, Broad Sanctuary, S.W. | *In- and Out-Surgeon*, Mr. Barrow, Tu. F. 9 |

### EYE DISEASES.

| Hospitals and Terms of Admission. | Physicians, Surgeons, and Medical Officers, with Days and Hours of Attendance. |
|---|---|
| Central London Ophthalmic Hosp., 238a, Gray's Inn Rd., W.C. Sec., Mr. G. H. Leah. Admission free | *Surgeons*, Messrs. T. Archer, G. Abbott, W. Charnley, W. Jessop, E. Clarke, J. R. Kemp and Granville Barker (House Surgeon). Daily, 1 |
| Evelina Hosp. for Sick Children, Southwark Bridge Rd. | *Surgeon*, Dr. W. Brailey, Th. 1 |
| Great Northern Central Hospital, Caledonian Rd., N. | *Surgeon*, Mr. Hutchinson, Jnr., Tu. F. 9.30 |
| Guy's Hospital, St. Thomas's Street, S.E. | *Surgeons*, Mr. Higgens, Dr. Brailey, Tu. F. 12.30 |
| Hospital for Sick Children, 49, Gt. Ormond Street, W.C. | *Surgeon*, Mr. R. Marcus Gunn, Tu. 2 |
| King's College Hospital, Portugal Street, W.C. | *Surgeon*, Mr. McHardy, M. W. Th. 1.30 |
| London Hospital, Whitechapel Road, E. | *Surgeons*, Messrs. Waren Tay, S. 9; Eve, Tu. 9 |
| Middlesex Hospital, Berners Street, W. | *Surgeon*, Mr. Lang, Tu. F. 9 |
| National Hosp. for the Paralysed, etc. Queen Sq., W.C. | *Surgeons*, Messrs. R. Brudenell Carter, F. 4; R. Marcus Gunn, Tu. 4 |
| North-West London Hosp. 18, Kentish Town Rd., N.W. | *Surgeon*, Dr. W. Collins, F. 3.30 |
| Paddington Green Children's Hosp., Paddington Grn. | *Surgeon*, Mr. W. H. H. Jessop, Th. 9 |
| Royal Free Hospital, Gray's Inn Road, W.C. | *Surgeon*, Mr. Mackinlay, M. F. 9 |
| Royal London Ophthalmic Hospital, Blomfield Street, Moorfields, E.C. Sec., Mr. R. J. Newstead. Admission free to the poor. | *Surgeons*, Messrs. J. W. Hulke, W. S.; G. Lawson, Tu. F.; J. Couper, Tu. F.; W. Tay, M. Th.; J. Tweedy, Tu. F.; E. Nettleship, W. S.; R. M. Gunn, W. S.; Lang, M. Th. Hours, 8 to 10 |
| Royal South London Ophthalmic Hosp., 6, St. George's Circus, S.E. Sec., Mr. C. Comyn. Free to the necessitous poor; or by payment | *Surgeons*, Messrs. Watson, McHardy. Daily, 2 |
| Royal Westminster Ophthalmic Hospital, King William Street, W.C. Sec., Mr. T. Beattie-Campbell. Poor and accidents free | *In- and Out-Surgeons*, Messrs. Power, M. F.; Frost, M. Th.; Rouse, Tu. S.; Hartridge, Tu. F.; Macnamara, M. Th.; Cowell, W. S.; Juler, W. S. Hour, 1 |
| St. Bartholomew's Hospital, Smithfield, E.C. | *Surgeons*, Messrs. Power, Th. 2; Vernon, Tu. S. 2.30 |
| St. George's Hospital, Hyde Park Corner, S.W. | *Surgeons*, Messrs. B. Carter, Frost, M. Th. 2; F. 1.15; W. S. 2 |
| St. Mary's Hospital, Cambridge Place, Paddington. W. | *Surgeons*, Messrs. A. Critchett, H. Juler, Tu. F. 9.30 |
| St. Thomas's Hospital, Westminster Bridge Road, S.E. | *Surgeons*, Messrs. Nettleship, Tu. Th. F. 1.30; Lawford, M. W. 1.30 |
| University College Hospital, Gower Street, W.C. | *Surgeon*, Mr. Tweedy, M. Tu. Th. F. 2 |
| West London Hospital, Hammersmith Road, W. | *Surgeons*, Messrs. B. Vernon, H. Dunn Tu. Th. S. 2 |
| Westminster Hospital, Broad Sanctuary, S.W. | *Surgeon*, Mr. Cowell, M. Th. 2 |
| Western Ophthalmic Hosp. 153 and 155, Marylebone Rd., W. Sec., Mr. G. E. Manton. By letter or small payments; free to poor | *Surgeons*, Drs. Miller, M.; Collins, F., Wheatly, M. Th.; Messrs. Carmalt Jones, W.; Buxton, Tu. F.; Nunn, W. S. Hour, 2 |

### The In- and Out-Patients' Hospital Diary.

### ORTHOPÆDIC CASES.

| Hospitals and Terms of Admission. | Physicians, Surgeons, and Medical Officers, with Days and Hours of Attendance. |
|---|---|
| City Orthopædic Hospital, 27, Hatton Garden, E.C. Sec., Mr. E. Dereuth. Admission free | *Surgeons*, Mr. E. J. Chance and House-Surgeon. M. Tu. Th. F. 2.30. New cases Tu. F. 2.30 |
| National Orthopædic Hospital for the Deformed, 234, Gt. Portland St., W. Sec., Mr. H. H. Canning. Free to poor; others by letter or payment | *In-Physician*, Dr. C. Beevor. *In- and Out-Surgeons*, Messrs. F. R. Fisher, M. Th. 2; W. J. Roeckel, Tu. F. 2; E. M. Little, W. 1 |
| Royal Orthopædic Hospital, 297, Oxford Street, W. Sec., Mr. B. Maskell. By Governor's letter | *Surgeons*, Messrs. B. E. Brodhurst, H. A. Reeves, C. Read, W. E. Balkwill, H. F. Baker. Daily, 1 |
| St. Bartholomew's Hospital, Smithfield, E.C. | *Surgeon*, Mr. Walsham, M. 2.30 |
| St. George's Hospital, Hyde Park Corner, S.W. | W. 2. *See* General Hospitals |

### PARALYSIS, EPILEPSY, AND DISEASES OF THE NERVOUS SYSTEM GENERALLY.

| | |
|---|---|
| Hospital for Diseases of the Nervous System, 32, Portland Terrace, Regent's Park Road, N.W. Sec., Mr. Howgrave Graham. By contributor's letter, or payment according to means, but free to necessitous poor | *In-Physicians*, Drs. J. Althaus, Th.; Hughes Bennett, M.; G. Ogilvie, Tu. Hour 2. *Out-Physicians*, Drs. Laycock, F.; F. Hawkins, W.; J. Althaus, Th.; G. Ogilvie, Tu.; H. Bennett, M. Hour, 2. *In- and Out-Surgeons*, Messrs. J. Bloxam, A. P. Gould, M. Tu. W. Th. F. 2 |
| National Hospital for the Paralysed and Epileptic, Queen Square, Bloomsbury, W.C. Sec. and Director, Mr. B. B. Rawlings. Most of the beds are reserved free to the necessitous poor; the others are for patients who contribute a Guinea a week | *In-Physicians*, Drs. J. S. Ramskill, Tu.; C. B. Radcliffe, M.; J. H. Jackson, F.; T. Buzzard, W. Hour, 2.30. *In-Surgeons*, Messrs. W. Adams, M. 3; V. Horsley, F. 2.30. *Out-Physicians*, Drs. T. Buzzard, W., H. C. Bastian, Tu.; W. R. Gowers, M.; D. Ferrier, F. 2.30; J. A. Ormerod, Tu. W.; C. E. Beevor, M; F. Hour, 2.30 |
| National Hospital for Diseases of the Heart and Paralysis, 32, Soho Sq., W. Sec., Capt. F. Handley. In patients require recommendation of Life Governor. | *In-Physicians*, Drs. V. Ambler and A. Duncan, M. W. Th. *In-Surgeon*, Mr. G. Bennett, Tu. *Out-Physicians*, Drs. V. Ambler, A. Duncan, R. Varley, J. Murray, M. 2, 7; Tu. 3; W. 2, 8; Th. 2; F. 3. *Out-Surgeon*, Mr. G. Bennett, Tu. 5 |
| West End Hospital for Diseases of the Nervous System, 73, Welbeck St., W. Hon. Sec., Mr. H. A. Dowell. Children only as in-patients. Out-patients by letter or payment. | *In- and Out-Physicians*, Drs. H. Tibbits, M. Th. 1.30; Stretch-Dowse, Tu. W. 1.30; S. H. Armitage, W. Tu. F. 6. *In- and Out-Surgeon*, Mr. Benson |

### DISEASES OF THE SKIN.

| | |
|---|---|
| Charing Cross Hospital, West Strand, W.C. | *Physician*, Dr. Sangster, M. 2 |
| Guy's Hospital, St. Thomas Street, S.E. | *Physicians*, Drs. Pye-Smith, Hale-White, Tu. 12 |
| Hospital for Diseases of the Skin, 52, Stamford St., S.E. Sec., Mr. S. Hayman. Free to the necessitous; also by payment | *Physician*, Dr. F. J. Payne. *Surgeons*, Messrs. J. Hutchinson, Waren Tay, and Wyndham Cottle. (Out-patients seen M. W. 3 Tu. Th. F. 2) |
| King's College Hospital, Portugal Street, W.C. | *Physician*, Dr. Duffin, F. 1 |
| London Hospital, Whitechapel Road, E. | *Physician*, Dr. S. Mackenzie, Th. 9 |
| Middlesex Hospital, Berners Street, W. | *Physician*, Dr. R. Liveing, Tu. 4 |
| North-West London Hosp. 18, Kentish Town Rd., N.W. | *Physician*, Dr. J. Stowers, Tu. 1.30 |
| Paddington Green Children's Hospital, Paddington | *Physician*, Dr. T. Colcott Fox, S. 9.15 |
| St Bartholomew's Hospital, Smithfield, E.C. | *Surgeon*, Mr. H. Cripps, F. 1.30 |

### DISEASES OF THE SKIN.—*Continued.*

| Hospitals and Terms of Admission. | Physicians, Surgeons, and Medical Officers, with Days and Hours of Attendance. |
|---|---|
| St. George's Hospital. Hyde Park Corner, S.W. | *Physician*, Dr. Cavafy, W. 1.30 |
| St. John's Hospital for Diseases of the Skin, 45, Leicester Sq., W.C. Branch, Markham Square, Chelsea, S.W. Sec., Mr. St. Vincent Mercier. In-patients by letter; out-patients free or by small payment | *Physicians*, Drs. Robinson, Dow, Harries, Campbell, Bourns. Daily 2; M. W. F. 7. Markham Sq. Branch, M. 10 and Th. 10 and 7. *Surgeon*, Mr. J. Milton, Tu. |
| St. Mary's Hospital, Cambridge Place, Paddington, W. | *Surgeon*, Mr. Morris, M. Th. 9.30 |
| St. Thomas's Hospital, Westminster Bridge Road, S.E. | *Physician*, Dr. Payne, W. 1.30 |
| University College Hospital, Gower Street, W.C. | *Physician*, Dr. Crocker, W. 1.30, S. 9.15 |
| Westminster Hospital, Broad Sanctuary, S.W. | *Physician*, Dr. T. C. Fox, W. 1.30 |

### STONE AND OTHER DISEASES OF URINARY ORGANS.

| | |
|---|---|
| London Hospital, Whitechapel Road, E. | Daily, 1 30. *See* General Hospitals |
| St. Peter's Hospital for Stone and Urinary Diseases, Henrietta Street, W.C. Sec., Mr. W. E. Scott. Admission free. Only women and children are seen on Fridays. Operation day, Wednesday | *In-Surgeons*, Messrs. W. J. Coulson, F. R. Heycock. *Out-Surgeons*, Messrs. E. H. Fenwick, M. 2, S. 4 to 7; F. S. Edwards, M. 5 to 7, Th. 2; Heycock, Tu. 2, W. 5 to 7, F. 2 |

### DISEASES AND IRREGULARITIES OF THE TEETH.

| | |
|---|---|
| Belgrave Hosp. for Children, 77 & 79 Gloucester St., Pimlico | *Surgeon*, Mr. G. W. Payne, W. 9 |
| Charing Cross Hospital, West Strand, W C. | *Surgeon*, Mr. Fairbank, M. W. F. 9.0 |
| Dental Hospital of London, Leicester Sq., W.C. Sec., Mr. J. F. Pink. Poor free. For special operation a Governor's letter is required | *Surgeons*, Messrs. Canton, Gregson, Hepburn, S. Bennett, Underwood, Woodhouse, Hern, Matheson, Parkinson, Read, Rogers, Truman. Daily, 9 to 11 |
| Evelina Hosp. for Sick Children, Southwark Brdg. Rd. | *Surgeon*, Mr. R. Denison Pedley, Tu. 9 |
| German Hospital, Dalston lane, Dalston, E. | *Surgeons*, Messrs. A. P. Reboul, C. West, Th. 10 to 11 |
| Great Northern Central Hospital, Caledonian Rd, N. | *Surgeon*, Mr. E. Keen, W. 1.30 |
| Guy's Hospital, St. Thomas Street, S.E. | *Surgeons*, Messrs. Moon, Pedley, Tu. Th. F. 12.30 |
| Hospital for Consumption, Brompton, S.W. | *Surgeon*, Mr. C. J. Noble, W., and when wanted |
| Hospital for Sick Children, Gt. Ormond Street, W.C. | *Surgeon*, Mr. Cartwright, W. 9 |
| King's College Hospital, Portugal Street, W.C. | *Surgeon*, Mr. Cartwright, Tu. F. 10 |
| London Hospital, Whitechapel Road, E. | *Surgeon*, Mr. A. W. Barrett, Tu. 9 |
| London Temperance Hosp., Hampstead Road, N.W. | *Surgeon*, Mr. Adolphus Alexander, M. 2 |
| Metropolitan Free Hospital, Gray's Inn Road, W.C. | *Surgeon*, Mr. G. Curnock, Tu. Th. S. 9 |
| Middlesex Hospital, Berners Street, W. | *Surgeons*, Messrs. Bennett, M. 9.30; W. 9; Hern, W. 9, F. 9.30 |
| National Dental Hospital, 149, Gt. Portland Street, W. Sec., Mr. A. G. Klugh. The poor, and all urgent cases admitted free; others by Subscriber's letter | *Surgeons*, Messrs. H. Weiss, W. Weiss, M.; A. Smith, G. Bradshaw, Tu.; G. A. Williams, M. Davis, W.; A. F. Canton, H. G. Read, Th.; T. Gaddes, W. S. Thomson, F.; H. Rose, W. R. Humby, S. Hours 9 to 11 |
| National Orthopædic Hospital, 234, Gt. Portland st, W. | *Surgeon*, Mr. H. Harris |

